# Perspectives on the Impact of Sampling Design and Intensity on Soil Microbial Diversity Estimates

**DOI:** 10.3389/fmicb.2019.01820

**Published:** 2019-08-07

**Authors:** Syrie M. Hermans, Hannah L. Buckley, Gavin Lear

**Affiliations:** ^1^School of Biological Sciences, The University of Auckland, Auckland, New Zealand; ^2^School of Science, Auckland University of Technology, Auckland, New Zealand

**Keywords:** soil bacteria, biogeography, biodiversity, national datasets, global datasets

## Abstract

Soil bacterial communities have long been recognized as important ecosystem components, and have been the focus of many local and regional studies. However, there is a lack of data at large spatial scales, on the biodiversity of soil microorganisms; national or more extensive studies to date have typically consisted of low replication of haphazardly collected samples. This has led to large spatial gaps in soil microbial biodiversity data. Using a pre-existing dataset of bacterial community composition across a 16-km regular sampling grid in France, we show that the number of detected OTUs changes little under different sampling designs (grid, random, or representative), but increases with the number of samples collected. All common OTUs present in the full dataset were detected when analyzing just 4% of the samples, yet the number of rare OTUs increased exponentially with sampling effort. We show that far more intensive sampling, across all global biomes, is required to detect the biodiversity of soil microorganisms. We propose avenues such as citizen science to ensure these large sample datasets can be more realistically achieved. Furthermore, we argue that taking advantage of pre-existing resources and programs, utilizing current technologies efficiently and considering the potential of future technologies will ensure better outcomes from large and extensive sample surveys. Overall, decreasing the spatial gaps in global soil microbial diversity data will increase our understanding on what governs the distribution of soil taxa, and how these distributions, and therefore their ecosystem contributions, will continue to change into the future.

The geographic ranges of biological species, and therefore the biodiversity of ecosystems, are continually changing over ecological and evolutionary timescales. The collation of national and international databases has proven vital to better understand patterns in current species distributions, supporting evidence-based conservation efforts (Jetz et al., 2012), and to predict species range-shifts under, for example, climatic change and future land use scenarios (Thomas et al., 2004; Pompe et al., 2008). However, although climate and land use projections are increasingly highly resolved, often at resolutions of a few kilometers or finer (Chen et al., 2015; Abatzoglou et al., 2018), the spatial grain of resolution for most known species distributions remains far coarser (Jetz et al., 2012). Microorganisms, the most abundant group of organisms on Earth, are key players in global biogeochemical cycles, yet only limited attempts have been made to characterize their distributions across wide geographic ranges using analyses of large datasets. This is especially true for soil microbial communities, where environmental heterogeneity leads to many distinct microbial habitats (Fierer, 2017), and global dissimilarities in soil physico-chemical characteristics present unique considerations to ensure accurate cataloging of their diversity across landscapes, regions and continents. Substantial efforts are required to reduce the gaps in soil microbial diversity data, which will require studies with adequate sampling depth across all global biomes.

Systematic surveys of microbial life are essential for providing new perspectives on bacterial distributions and the causal processes driving these patterns. Understandably, the significant effort and costs associated with consistently sampled national or global studies means it is common to see research that covers large spatial extents, but with spatially irregular sampling and relatively low replication. Even the most extensive national-scale datasets of soil bacterial biogeography, such as surveys of the British Isles (Griffiths et al., 2011) and Australia (Bissett et al., 2016), use non-uniform sampling designs, and may comprise of sample replication that is biased toward more populated and/or accessible areas. To avoid or account for these biases, random or regular (e.g., grid-based) sampling is considered desirable, but is rarely attempted (Powell et al., 2015; Terrat et al., 2017). Therefore, to inform approaches for expanding global soil microbial datasets, it is useful to understand the effects of these alternative sampling approaches on our estimations of bacterial soil community structure.

Comprehensive global soil microbial biodiversity datasets must be assembled from regional studies; however, the relative comparability and compatibility of regional datasets will determine how useful a given global dataset would be. Thus, here, we explore a dataset that does not suffer from the usual sampling limitations of many regional datasets in the published literature to determine the possible effects of variation in sampling design and replication on detection of soil microbial biodiversity. Using bacterial community data collected across a 16-km regular sampling grid within France as part of the French Soil Quality Monitoring Network (Ranjard et al., 2010; Terrat et al., 2017), we quantified the effects of sampling strategy and intensity for soil bacterial biodiversity estimates (see Supplementary Appendix S1). This analysis shows that the most common OTUs were, in fact, detectable from the analysis of only ∼4% samples collected (Figure 1A, as indicated by the plateau of the curve). This is largely irrespective of whether samples were collected from random locations, in a regular grid format, or proportionally to represent the natural diversity of soil environments (Figures 1B, 2). This pattern held true, even if a geographic subset of the dataset was analyzed (Supplementary Appendix S3 and Supplementary Figure S2). The dominance of a relatively small number of bacterial taxa is similarly reported at the global scale (Delgado-Baquerizo et al., 2018b). Variation in sampling design and intensity that is commonly observed among regional datasets may therefore not be an important consideration for capturing common and dominant bacterial taxa at a global scale.

**FIGURE 1:**
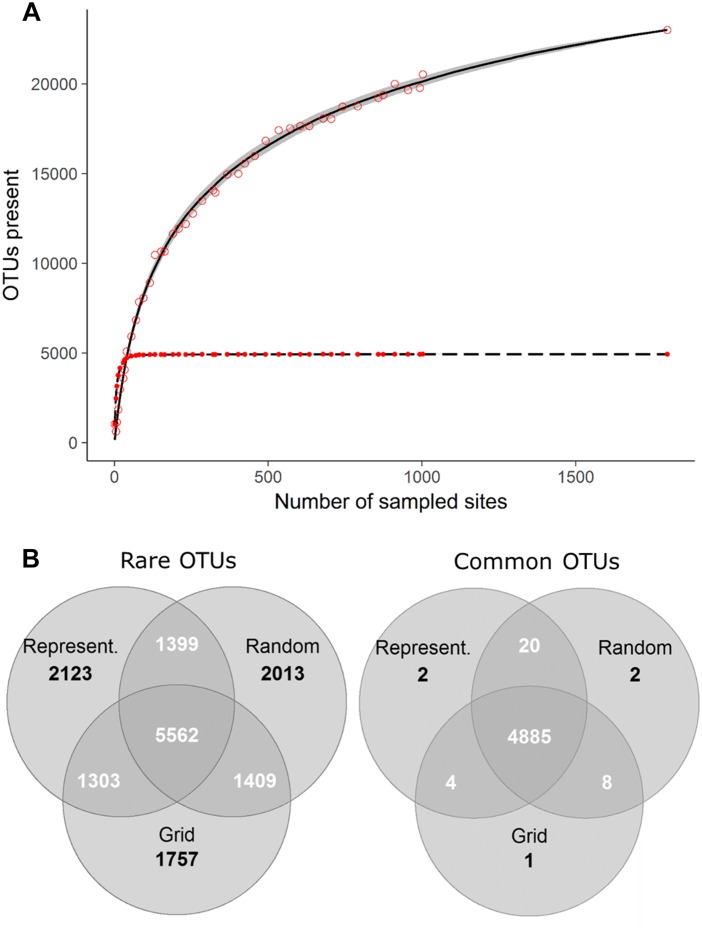
**(A)** Taxa accumulation curve showing the OTUs detected by the random (lines) and grid (points) sampling approaches. The lines indicate the number of rare (>0.001% of total reads; solid line) and common (<0.001% of total reads; dashed line) OTUs detected with increased random sampling; 100 permutations were used, with sites added in a random order, to calculate average values. Standard deviations are indicated in gray. Red points indicate the number of rare (hollow points) and common (filled points) OTUs detected with decreasing grid size (and therefore increased sampling intensity). **(B)** The number of unique and shared OTUs detected by the different sub-sampling approaches.

**FIGURE 2:**
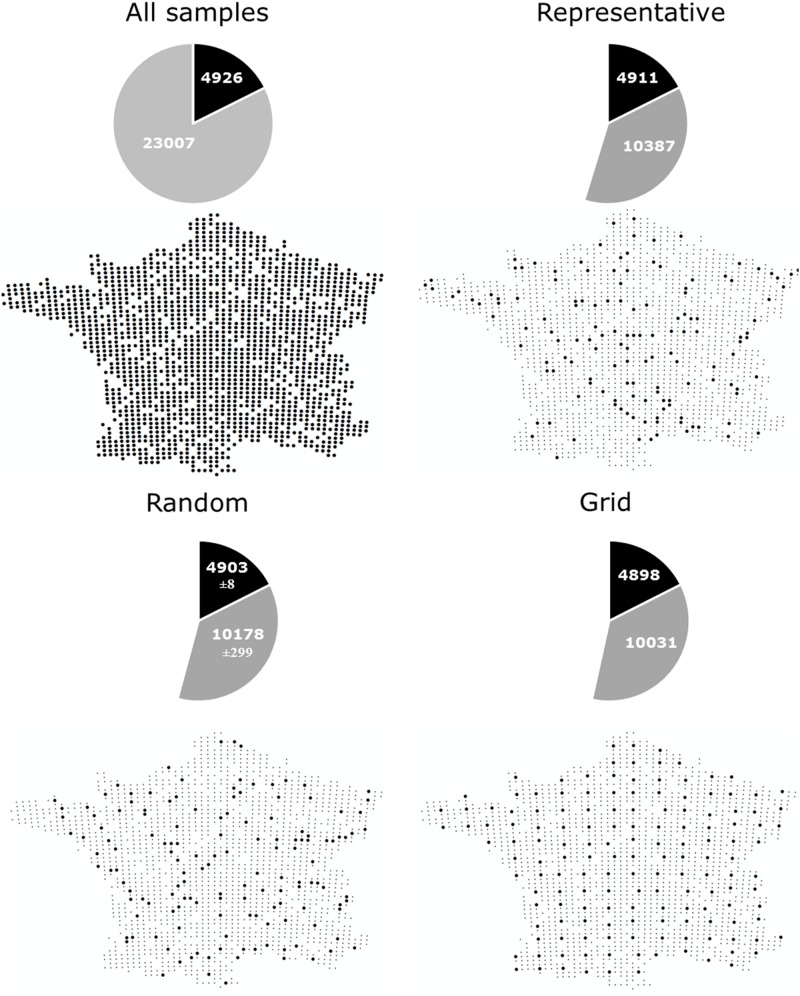
The number of common (> 0.001% of total reads) and rare (< 0.001% of total reads) OTUs captured by different sampling approaches; All samples: Locations of samples comprising the complete dataset which we subsampled, containing 1798 samples collected on a 16 km grid (Terrat et al., 2017), Representative: Sampling described by Orgiazzi et al. (2018) to capture a range of different land uses, soil properties and climatic conditions (*n* = 144), Random: 144 samples randomly selected from the complete dataset (100 permutations were used and the average ± standard deviation is given), Grid: 151 samples collected in an approximate grid format.

While intensive sampling of local environments may not be required for the detection of many common taxa, sampling intensity significantly impacts community diversity measures, largely caused by the increased detection of rare OTUs with greater sampling effort (Figure 1A). Taxa may be rare due to their low local abundance, habitat specificity or restricted geographic spread, but can have a disproportionately large influence on ecosystem processes (Jousset et al., 2017; Karimi et al., 2018). Conditionally rare bacterial taxa (Shade et al., 2014) may be more metabolically active, even when present in low abundance (Dimitriu et al., 2010), and their vast genetic resource has been shown to enhance the functionality of more abundant microbes, via the horizontal transfer of beneficial genes (Low-Décarie et al., 2015). The number of rare OTUs detected in the French dataset did not appear to be influenced by sampling design (i.e., grid vs. random sampling) but did increase with increasing sample numbers (Figure 1A). Even with the inclusion of all available samples, a complete plateau in the increase of rare OTUs detected was not reached, although the number of new OTUs detected did decrease with increasing sample numbers (Figure 1A). This suggests that decisions about sampling intensity within national biodiversity monitoring are crucial for generating datasets that will be globally comparable where the distributions of rare taxa are of interest.

Perhaps the most comprehensive and coordinated effort to catalog microbial diversity across a range of environments around the globe is the Earth Microbiome Project (EMP; (Gilbert et al., 2014), highlighting the substantial progress that can be made through cooperative research. However, even within the EMP, multiple regions are grossly under-sampled and poorly represented (Supplementary Appendix S2 and Supplementary Figure S1); the continents of Europe, Asia, South America, Africa and Australia are each represented by ≤10 spatially independent samples of microbial DNA. Similar spatial biases are evident in the study of Delgado-Baquerizo et al. (2018b; see Supplementary Appendix S1 and Supplementary Figure S1 in their article). Adding to this knowledge that the spatial scaling of variation in microbial community structure differs widely across spatial scales (Constancias et al., 2015), substantial efforts must be made to further reduce global gaps in soil bacterial diversity data. Sampling to proportionally represent the relative diversity of different soil environments, or even to over-represent rare environments, or conditions, may be required for valid statistical analysis at global scales, since different environmental gradients dominate community assembly across different biomes and spatial scales. For example, soil pH is often strongly correlated with bacterial diversity (Thomson et al., 2015), to the extent that it can be used to generate global predictions of bacterial diversity (Griffiths et al., 2015). However, there are certain biomes where this is not true, such as grasslands where instead aridity drives bacterial diversity (Maestre et al., 2015). Such findings highlight the importance of conducting surveys of microbial life appropriate for data analysis at multiple scales because understanding of what affects bacterial community composition at small scales cannot necessarily be extrapolated to make reliable conclusions at larger scales. Grid-based sampling designs are the most statistically powerful way to achieve this, providing that the resolution of the grid is finer than the scale processes of interest (Hirzel and Guisan, 2002; Mallarino and Wittry, 2004; Nanni et al., 2011).

Increasing the size of national and international soil microbial datasets can be achieved by increased cooperation among research facilities, and perhaps even between researchers and the general public. Taking a leaf out of the macro-organism ecologist’s handbook, pursuing a “citizen science” approach is considered particularly useful for the collection of samples from more remote areas (Bahls, 2014), although consistent and well documented sample treatments must be ensured to allow accurate comparability and reproducibility (Dickie et al., 2018). There are already many examples studies where the public has been engaged to help collect data for ecological surveys of birds, trees and tropical reef species (Mckenzie et al., 2007; Ockendon et al., 2009; Roelfsema et al., 2016). Arguably, collecting and transporting soil samples requires much less time and expertise than identifying and monitoring animals and plants. Since public engagement in macro-organism surveys has been shown to be a successful biodiversity monitoring tool (Devictor et al., 2010), and is increasingly being utilized for soil microbial surveys (e.g., microblitz^1^), this is an avenue worth exploring to increase global coverage of bacterial community data.

Ensuring better sampling designs and global coverage alone will not be sufficient; ecologists are increasingly interested in understanding the factors affecting the present day distributions of organisms. This requires microbial DNA to be collected in tandem with a suite of relevant physicochemical variables; however, a shortcoming of many of large-scale studies published to date is the limited range of metadata collected, as the high costs associated with exhaustive soil analyses remains a major obstacle. A notable workaround for this problem is where microbial surveys are partnered with soil physicochemical monitoring programs (Dequiedt et al., 2011; Griffiths et al., 2011; Ranjard et al., 2013; Hermans et al., 2017) which include a comprehensive list of soil nutrients, physical characteristics and heavy metal concentrations. The benefits of collecting biodiversity data alongside traditional large scale soil monitoring programs is increasingly being recognized (Orgiazzi et al., 2018). As environmental monitoring agencies become more aware of the utility for microbial data to report on the health and production potential of diverse environments, existing monitoring programs are increasingly likely to be adapted to provide valuable support for microbiological investigations, helping to identify key correlates associated with changes in community composition and taxon presence across diverse spatial and temporal scales.

Microbial ecologists have tended to describe changes in composition and diversity from DNA sequence data, often without naming individual taxa, or even groups of bacteria. Arguably, this approach has inhibited our understanding of the natural history of bacteria (Martiny and Walters, 2018). However, unlike DNA fingerprinting methods, which previously dominated large-scale molecular assessments of microbial community diversity (Gobet et al., 2014), next-generation sequencing (NGS) allows taxa to now be identified from their unique DNA barcodes and grouped at various taxonomic levels. It is essential we go beyond describing general changes in microbial community composition, to looking at individual taxa, or phylogenetic or functional groups of taxa, in more detail, in the same way that traditional ecologists studying plants and animals characterize biodiversity by describing and naming the species present (Fierer, 2017; Martiny and Walters, 2018). Encouragingly, with more paired microbial and metadata being collected, NGS technologies are beginning to be used to assess not only taxonomic data, but also to make predictions of microbial functional community attributes. The expense associated with adequately sequencing complex soil metagenomes using shotgun DNA approaches mean that although microbial functional diversity has been assessed under different biomes and land uses (Fierer et al., 2012; Mendes et al., 2015), coordinated efforts to collect metagenome data from large scale soil datasets remain extremely limited. Nevertheless, scientists can capitalize on the increased availability of soil taxonomic and associated metadata to make informed predictions of the biogeography of microbial taxa and traits. As the spatial extent and grain of soil microbial community surveys increases, the relationship between soil variables such as pH, and concentrations of nutrients or potential pollutants and the distribution and relative abundance of microbial taxa are becoming better understood (Hermans et al., 2017; Karimi et al., 2018). This allows ever stronger predictions to be made regarding the environments where specific organisms or groups of organisms might be found (Delgado-Baquerizo et al., 2018a), even for organisms that are yet to be cultured or are only known from their 16S rRNA sequences.

Rapidly improving molecular methods means we also need to consider how samples collected today can be used with technology that may not yet be available, or financially achievable, for use in large scale biodiversity monitoring methods. Technological changes are very likely to occur for how extracted DNA (or RNA) is analyzed, but improvements and changes may also occur in how raw sample material is processed. For example to extract genetic material. DNA extraction biases have repeatedly been shown to exhibit biases and limitations for different sample and organisms types (Luna et al., 2006; Wagner Mackenzie et al., 2015; Hermans et al., 2018). Future improvements to current DNA extraction techniques, or the development of new methods, could lead to desires to re-analyze previous samples to obtain more accurate representations of the microbial communities that were present. It has previously been shown that bacterial DNA can be extracted from dried soil samples over a century after the soil was stored (Clark and Hirsch, 2008), and that DNA can be maintained for months at −80°C (Gorokhova, 2005). However, more research needs to be conducted to determine the effect of time and storage conditions on microbial community composition in raw sample material, and the degradation of DNA over years, rather than months. Following current best practice storage methods for the large sample numbers that will be generated by national, and global surveys of microbial diversity is essential. This will provide not only a ‘snapshot in time’ of the current biodiversity of soil bacteria globally, but also allow the application of future biodiversity monitoring methods without repeating the labor intensive, and expensive sampling process.

Significant progress has been made in the last decade to catalog microbial diversity across the globe, yet the lack of systematic approaches for sampling across national and global scales, is leading to unbalanced datasets which are failing to cover all of the planet’s biomes. Greater coordination among researchers, collaboration with soil monitoring agencies and the general public, could facilitate the collection of more spatially extensive and -intensive datasets. Extensive sampling of soils across the globe, to identify the microbial taxa residing within them and their functions, is essential to increase our understanding of natural variation in these communities, the effect that human land use has on microorganisms, and the impact that climatic change may have on future ecosystem function.

## Author Contributions

SH analyzed the data. All authors contributed to the writing of the manuscript.

## Conflict of Interest Statement

The authors declare that the research was conducted in the absence of any commercial or financial relationships that could be construed as a potential conflict of interest.

## Footnotes

1 www.microblitz.com.au
