# Supplementary material for: Perspectives on the Impact of Sampling Design and Intensity on Soil Microbial Diversity Estimates
Source: Front Microbiol. 2019 Aug 7;10:1820. doi: 10.3389/fmicb.2019.01820 (PMC6692435; doi:10.3389/fmicb.2019.01820)
Supplement: Supplementary file 1 [file Data_Sheet_1.docx]

Supplementary Material

# Appendix 1: Methods

## Data

Raw sequence reads (.sff files) from the Terrat et al. (2017) study were downloaded from the EBI database system (in the Short Read Archive) under project accession PRJEB21351, along with oligo tag information and site coordinates. A total of 69 files were downloaded, corresponding to 69 GS FLX Titanium sequencing runs which contained between 3 to 30 multiplexed samples. Given the differences in sequence length and depth obtained with more modern sequencing technologies such as Illumina sequencing, it is possible that different platforms will produce slightly different results, however it has been shown that these methods generally produce consistent results (Claesson et al., 2010; Trembley et al., 2015)

## Bioinformatics

Denoising, demultiplexing and quality filtering of raw sequence reads was done in mothur v1.39.5 as per the standard operating procedure for 454 data (Schloss et al., 2011), with all recommended or default parameters. This resulted in 1,798 samples for subsequent analyses.

USEARCH v9.0.2132 was used to dereplicate the cleaned sequence data and remove singletons (sequences which occurred only once across the entire dataset). OTU clustering was performed using the *cluster_otus* command, using the default 97% similarity threshold. This step also removes chimeric sequences; while 100% removal of chimeric sequences cannot be guaranteed this approach has been shown to be highly effective for 454 data (Edgar, 2013).

## Subsampling and analyses

The OTU table from USEARCH was rarefied in R v 3.4.4 (R Core Team 2018) using the vegan package (Oksanen et al., 2018) to the minimum number of reads per sample in the dataset (5,529 reads). At this sequencing depth, the samples contained between 413 and 1604 OTUs each (1133 on average).

Three subsampling methods were used to determine the impact that different sampling approaches have on the measured biodiversity.

Representative sampling: Sampling described by Orgiazzi et al. (2018) to capture a range of different land uses, soil properties and climatic conditions across France. Samples from the Terrat et al. (2017) study which were in closest proximity to the sampling sites identified by Orgiazzi et al. (2018) were selected for this method (n=144).

Random sampling: The same sampling depth as achieved by the representative sampling (n=144) but samples were randomly selected from the 1,798 samples in the total dataset. This was repeated 10 times to obtain an average number of OTUs detected using this approach.

Grid: Approximately the same sampling depth as achieved by the representative sampling (n=151) but samples were selected from the total dataset in a grid format. The grid was created using ArcGIS v10 and samples from the Terrat et al. (2017) dataset which fell closest to the centre of each square in the grid were selected.

The OTUs in the full dataset (all 1,798 samples) were classified as either rare, or common, based on their total abundance (OTUs with <0.001% of reads across the dataset are considered rare, those with <0.001% of all reads are considered common). This threshold was deemed most appropriate as it classified only OTUs which were highly abundant (both in terms of abundance per site, as well as number of sites in which it occurred) as common. The threshold resulted in 23,007 OTUs classified as rare, and 4,926 OTUs classified as common.

The number of these rare and common OTUs obtained by each subsample was calculated, and Venn Diagrams were created to show the number of shared and unique OTUs detected by each approach. A taxa accumulation curve, showing the impact of sampling depth (number of samples) on the number of OTUs detected, was created using the ‘specaccum’ function in the vegan package (Oksanen et al., 2018), with default parameters for method=‘random’.

Grids ranging in size from 1 to 1122 samples were created in ArcGIS v10, and samples from the Terrat et al. (2017) dataset which fell closest to the centre of each square in the grid were selected. The total richness (number of OTUs) for each of the different sized grids was then calculated and overlaid on the taxa accumulation curve created as described above.

Overall, the results obtained by analyzing the full French dataset, as described above, are consistent with results obtained when analyzing a smaller region of the French dataset (see Appendix 3).

## References

Claesson, M. J., Wang, Q., O'sullivan, O., Greene-Diniz, R., Cole, J. R., Ross, R. P., & O'toole, P. W. (2010). Comparison of two next-generation sequencing technologies for resolving highly complex microbiota composition using tandem variable 16S rRNA gene regions. Nucleic acids research, 38(22), e200.

Edgar, R. C. (2013). UPARSE: highly accurate OTU sequences from microbial amplicon reads. Nature methods, 10(10), 996.

Jari Oksanen, F. Guillaume Blanchet, Michael Friendly, Roeland Kindt, Pierre Legendre, Dan McGlinn, Peter R. Minchin, R. B. O'Hara, Gavin L. Simpson, Peter Solymos, M. Henry H. Stevens, Eduard Szoecs and Helene Wagner (2018). vegan: Community Ecology Package. R package version 2.5-3. https://CRAN.R-project.org/package=vegan

R Core Team (2018). R: A language and environment for statistical computing. R Foundation for Statistical Computing, Vienna, Austria. URL https://www.R-project.org/

Schloss PD, Gevers D, Westcott SL. (2011). Reducing the effects of PCR amplification and sequencing artifacts on 16S rRNA-based studies. PloS ONE. 6:e27310. Accessed May 2018.

Terrat S, Horrigue W, Dequietd S, Saby NPA, Lelièvre M, Nowak V, et al. (2017) Mapping and predictive variations of soil bacterial richness across France. PLoS ONE 12(10): e0186766. https://doi.org/10.1371/journal.pone.0186766

Tremblay, J., Singh, K., Fern, A., Kirton, E. S., He, S., Woyke, T., ... & Tringe, S. G. (2015). Primer and platform effects on 16S rRNA tag sequencing. Frontiers in microbiology, 6, 771.

# Appendix 2: EMP Dataset

A total of 243 soil samples were analysed as part of the Earth Microbiome Project with unique latitude/longitude information as identified using the R code below and as shown in Supplementary Figure 1.

library(rworldmap)

library(ggplot2)

library(rgeos)

#plot the map

pdf(file="worldmap.pdf")

worldmap <- getMap(resolution = "low")

plot(worldmap, asp = 1.5)

#load in the data

metadata <- read.csv("emp_qiime_mapping_qc_filtered.tsv", sep = "\t", header = TRUE)

#remove samples with missing location values

metadata <- metadata[complete.cases(metadata$latitude_deg), ]

#keep only one sample per unique latitude/longitude combination

metadata <- metadata[!duplicated(metadata$latitude_deg) & !duplicated(metadata$longitude_deg), ]

#Just select only the data for mapping originating from ‘soil’

Soil<-subset(metadata, env_material =="soil")

#plot the points on the map

points(Soil$longitude_deg, Soil$latitude_deg, pch = 21, col = "darkred")

dev.off()


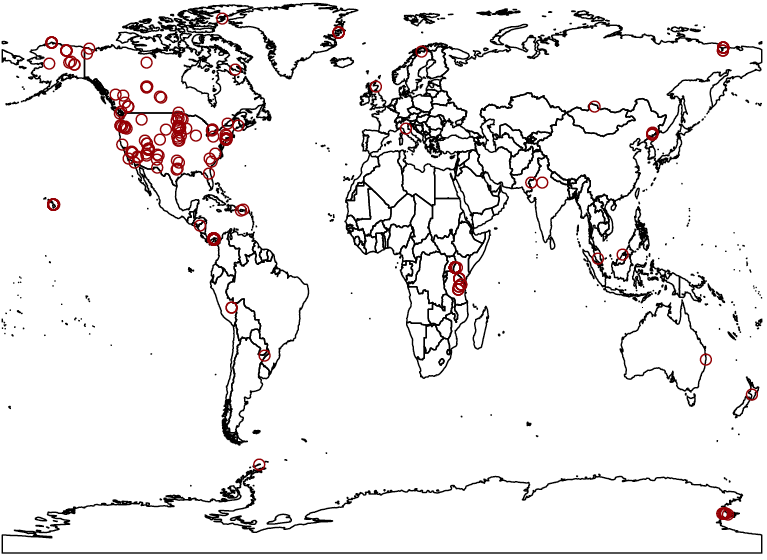


**Supplementary figure 1.** Location of sites from which soil microbial data were analysed as part of the Earth Microbiome Project as described in Thompson, L. R., Sanders, J. G., McDonald, D., Amir, A., Ladau, J., Locey, K. J., et al. (2017). A communal catalogue reveals Earth’s multiscale microbial diversity. *Nature* 551, 457–463. doi:10.1038/nature24621.

# Appendix 3: Supplementary results

When analyzing only the North-Eastern portion of the French dataset (Supplementary figure S2a), we find that, consistent with the results obtained when analyzing the full dataset (Figures 1 and 2, main text), only a very small number of samples is required to detect the dominant OTUs (Supplementary figure S2b). The number of rare OTUs detected, however, increased with increased sampling depth. There number of rare and dominant OTUs detected did not change when simulating random, representative or grid sampling of the North-Eastern portion of France.


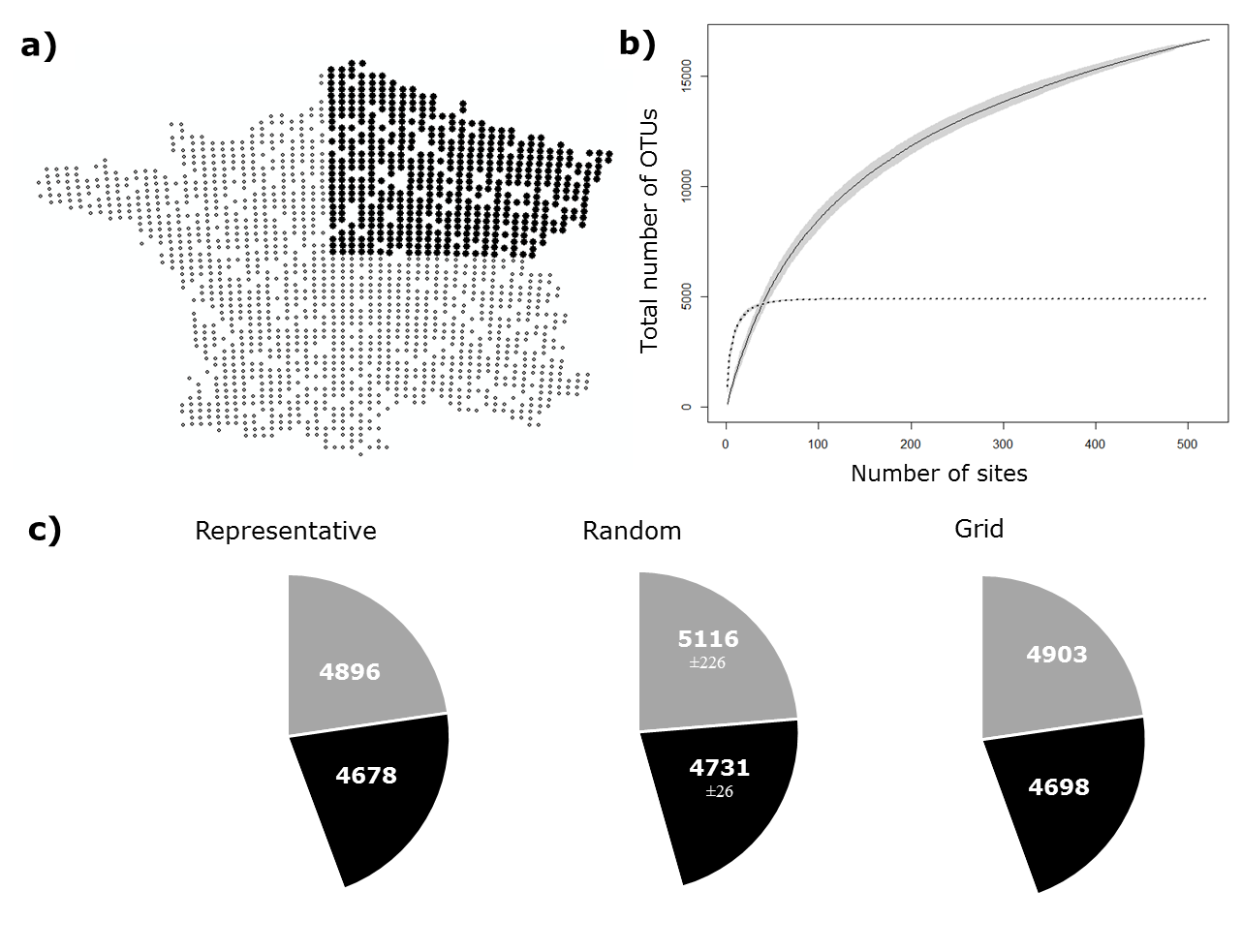


Supplementary figure 2: (a) The location of samples that were considered to be located in the North-Eastern portion of France (black dots). (b) Taxa accumulation curve showing the OTUs detected by increased sample numbers. The lines indicate the number of rare (>0.001% of total reads; solid line) and common (<0.001% of total reads; dashed line) OTUs detected with increased random sampling; 100 permutations were used, with sites added in a random order, to calculate average values. Standard deviations are indicated in grey. (c) The number of common (⬤) and rare (⬤) OTUs captured by different sampling approaches, presented as a fraction of the total OTUs present in the NE portion of the French dataset; Representative: Sampling described by Orgiazzi et al. (2018) to capture a range of different land uses, soil properties and climatic conditions (n = 44), Random: 44 samples randomly selected from the complete dataset (100 permutations were used and the average ± standard deviation is given), Grid: 44 samples collected in an approximate grid format.
